# Height outcome of the recombinant human growth hormone treatment in Turner syndrome: a meta-analysis

**DOI:** 10.1530/EC-18-0115

**Published:** 2018-03-26

**Authors:** Ping Li, Fei Cheng, Lei Xiu

**Affiliations:** Department of EndocrinologyBeijing Shijitan Hospital, Capital Medical University, Beijing, China

**Keywords:** recombinant human growth hormone, Turner syndrome, oxandrolone, height outcome, meta-analysis

## Abstract

**Objective:**

This study sought to determine the effect of the recombinant human growth hormone (rhGH) treatment of Turner syndrome (TS) on height outcome.

**Methods:**

We searched in MEDLINE, EMBASE and Cochrane Central Register of Controlled Trials and Cochrane Database of Systematic Reviews. A literature search identified 640 records. After screening and full-text assessment, 11 records were included in the systematic review. Methodological quality was assessed using the Cochrane Risk of Bias tool. RevMan 5.3 software was used for meta-analysis. We also assessed the quality of evidence with the GRADE system.

**Results:**

Compared with controls, rhGH therapy led to increased final height (MD = 7.22 cm, 95% CI 5.27–9.18, *P* < 0.001, *I*2 = 4%; *P* = 0.18), height standard deviation (HtSDS) (SMD = 1.22, 95% CI 0.88–1.56, *P* < 0.001, *I*2 = 49%; *P* = 0.14) and height velocity (HV) (MD 2.68 cm/year; 95% CI 2.34, 3.02; *P* < 0.001, *I*2 = 0%; *P* = 0.72). There was a small increase in bone age (SMD 0.32 years; 95% CI 0.1, 0.54; *P* = 0.004, *I*2 = 73%; *P* = 0.02) after rhGH therapy for 12 months. What is more, the rhGH/oxandrolone combination therapy suggested greater final height (MD 2.46 cm; 95% CI 0.73, 4.18; *P* = 0.005, *I*2 = 32%; *P* = 0.22), increase and faster HV (SMD 1.67 cm/year; 95% CI 1.03, 2.31; *P* < 0.03, *I*2 = 80%; *P* < 0.001), with no significant increase in HtSDS and bone maturation compared with rhGH therapy alone.

**Conclusions:**

For TS patients, rhGH alone or with concomitant use of oxandrolone treatment had advantages on final height.

## Introduction

Turner syndrome (TS) affects about one in 1500–2500 live-born females (1, 2). TS is a genetic disorder characterized by short stature, gonadal dysgenesis, cardiac and renal abnormalities and a particular neurocognitive profile of normally developed language abilities and impaired visual-spatial and/or visual-perceptual abilities. TS is the result of (partial) absence of one X-chromosome. As a chromosomal condition, there is no cure for Turner syndrome. Short stature is a common feature of TS, untreated women are approximately 20–21 cm shorter than normal women within their respective populations (3, 4, 5, 6, 7, 8).

Recombinant human growth hormone (rhGH) has been shown to increase growth and final height in girls who have Turner syndrome (9, 10, 11). rhGH therapy in Turner’s syndrome was initiated in 1983 (12). Oxandrolone (OX) is a synthetic non-aromatizable anabolic steroid with weak virilizing effects compared with testosterone, which has been used to increase adult height in TS (13, 14, 15, 16).

Treatment with rhGH, alone or in combination with the anabolic steroid oxandrolone, has been recommended for children with TS to improve final height. However, final height benefit remains uncertain. Some studies have been reported with optimistic responses to rhGH treatment alone (10, 17) or with the concomitant use of oxandrolone (18). However, rhGH alone or in combination with oxandrolone was estimated to have no effect on adult height in some studies (19, 20, 21). To date, whether rhGH alone or in combination with oxandrolone are effective in increasing height is still somewhat controversial. In addition, how much height may be gained in patients with TS is also an important consideration.

The aim of the present study was to evaluate height outcome of rhGH alone or with concomitant use of oxandrolone treatment in patients with TS, by using the meta-analytic approach as previously described.

## Methods

### Literature search

A comprehensive search of several databases from each database’s inception to March 30th, 2017, any language was conducted. The databases included PubMed, MEDLINE, EMBASE, Cochrane Central Register of Controlled Trials (CENTRAL) and Cochrane Database of Systematic Reviews. The search strategies were (growth hormone or somatotropin or somatropin or somatotrophin or somatrophin or Pituitary Growth Hormone or Recombinant Growth Hormones) and (Turner syndrome or Ullrich-Turner Syndrome or Bonnevie Ullrich Syndrome or Turner’s syndrome) and publication type (randomized controlled trials) as limiter. We also searched the reference list of all published original articles and several review articles we found for additional references. Publications arising from the same study group on the same patient cohort were considered as a single study for the purpose of this analysis. The study of this protocol-based review was consistent with the PRISMA statement.

### Inclusion and exclusion criteria

Studies were considered eligible if they met the following criteria: (1) randomized controlled trials (RCTs); (2) the participants had to have TS confirmed by karyotype; (3) the active intervention was rhGH: that is, biosynthetic human growth hormone, with a sequence identical to that of human growth hormone, marketed under any brand name; (4) intervention and comparison, rhGH alone vs placebo/no treatment, rhGH plus oxandrolone combination therapy vs rhGH alone therapy. We excluded uncontrolled studies, case series, cross-sectional studies and studies with short follow-up duration of less than 1 year. Only full-text articles were included; restriction was placed on the language of studies published in English.

### Primary and secondary outcomes

Primary outcomes were final height (final adult height is defined as the height at which epiphyses are closed or height velocity is less than 1 cm/year.) and height standard deviation (HtSDS) (height is often reported in standard deviations relative to a normal population or a population with TS.). Height velocity (HV), defined as increment in height per year in centimeters, and bone age (a measure of skeletal maturity) were secondary outcome measures of this study. Data were abstracted by a single reviewer and checked by a second reviewer.

### Data extraction, synthesis and statistical analysis

Two investigators (P L and F C) independently reviewed the articles and selected eligible studies according to the inclusion criteria for eligible studies. Irrelevant studies were excluded. Detailed information from each included studies was recorded by two authors independently. The following information was extracted from each study: name of first author, year of publication, country in which the study was conducted, number of participants of treatment and control group, initial height, final height, HtSDS, bone age and height velocity. Data extraction was done by two reviewers (P L and F C) with any disagreements resolved through discussion with a third reviewer (L X).

Data were summarized statistically if they were available, sufficiently similar and of sufficient quality. All data should be expressed as means with standard deviations (s.d.). When information was reported for more than one subpopulation in one study, each subpopulation was treated as a separate comparison in our meta-analysis. A random-effects model was employed to estimate weighted mean differences for HtSDS due to its different assays, calculated as the mean difference between groups. The heterogeneity of the included studies was evaluated with the Cochran Q and the *I* ^2^ statistic. For the Q statistic, *P* < 0.10 was considered statistically significant for heterogeneity. The *I* ^2^ statistic was assessed using the following ranges as guidelines: no heterogeneity (*I* ^2^ 0–25%); moderate heterogeneity (*I* ^2^ 25–50%); large heterogeneity (*I* ^2^ 50–75%) and extreme heterogeneity (*I*2 75–100%) When either Q statistics (*P* < 0.1) or *I* ^2^ statistic (>50%) indicated heterogeneity between studies, the random-effects model was preferred. Otherwise, the fixed-effect model was recommended. Statistical analyses were performed using RevMan 5.3 software.

### Risk of bias assessment

Two authors (F C and L X) independently assessed the risk of bias. Risk of bias was assessed by using the Cochrane Collaboration’s tool 15. Each study was assessed and scored as ‘high’, ‘low’ or ‘unclear’ risk of bias to the following criteria: random sequence generation; allocation concealment; blinding of participants and personnel; blinding of outcome assessment; incomplete outcome data; selective reporting and other bias. Blinding of patients and clinicians was extremely difficult and generally not feasible in these trials, and we judged that the primary outcome was less prone to be influenced by lack of blinding. Therefore, studies with high risk of bias for any one or more key domains except blinding were considered as at high risk of bias; while studies with low risk of bias for all key domains except blinding were considered as at low risk of bias; otherwise they were considered as at unclear risk of bias. Detailed method for the assessment of risk of bias is described in Fig. 4.

### Quality of evidence assessment

Quality of evidence was assessed per outcome, independently by two individuals (P L and L X), using the GRADE guidelines for rating the quality of evidence (22, 23, 24, 25, 26, 27, 28, 29, 30). We used the GRADE method to summarize the evidence profile regarding inconsistency, indirectness, imprecision and other sources of bias. GRADE criteria were used to downgrade the quality of evidence based on specific parameters. The quality of evidence for each outcome was rated as high, moderate, low or very low.

## Results

### Search results

**Figure 1:**
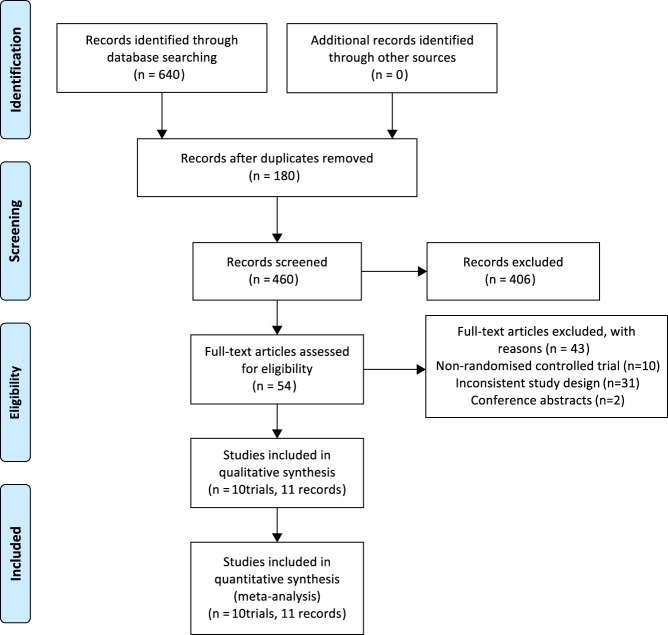
Flow diagram of literature search.

The initial searches identified 640 articles. A total of 586 publications were excluded based on review of the title and abstract for several reasons, including study type (case report, review or non-interventional study), population (children) or short duration of GH treatment (<12 months). Of the remaining 54 potentially relevant publications, 10 studies were non-randomized controlled study, 2 articles were conference abstracts, 31 of them do not meet our inclusion criteria. After all, 11 publications remained that described the results of 9 trials that met the clinical inclusion criteria (Fig. 1). Nine trials were published individually (10, 13, 16, 17, 31, 32, 33, 34, 35) and the results of 1 others were compiled in 2 publications (14, 36). Baseline characteristics of included RCTs were shown in Table 1.

**Table 1:** Characteristics of included studies.

Author	Publication	Design	Country	Interventions (dose)	Controls (dose)	Patients, no.	Duration of treatment (years)	Age at starting GH (year)	Age at starting estrogen (year)	Outcome measures
Davenport, M L	2007	RCT, open label	USA	rhGH (0.35 mg/kg/week)	No treatment	89	2	0.75~4	NA	HtSDS, bone age
Stephure, D K	2005	RCT	Canada	rhGH (0.30 mg/kg/week)	No treatment	154	5.7	7~13	13	HtSDS, bone age, height
Menke, L A	2010	RCT	Netherlands	rhGH (0.43 mg/kg/week) + oxandrolone (0.03 mg/kg/day) and rhGH + oxandrolone (0.06 mg/kg/day)	rhGH + placebo	133	5.8~6.7	2~15.99	12~16	HtSDS final height
Quigley, C A	2002	RCT open label	USA	rhGH (0.27 mg/kg/week) + placebo rhGH (0.27 mg/kg/week) + estrogen rhGH (0.36 mg/kg/week) + placebo rhGH (0.36 mg/kg/week) + estrogen	Placebo + placebo	232	1.5	6.9~12.5	13.5	HV
Ross, J L	2011	RCT	USA	rhGH (0.3 mg/kg/week) + placebo rhGH (0.3 mg/kg/week) + estrogen	Placebo + placebo, Placebo + estrogen	149	3~10.1	5~12.5	12	HtSDS, bone age, height
Job, J C	1991	RCT	France	rhGH (0.23 mg/kg/week) + oxandrolone (0.06 mg/kg/day)	rhGH	22	3	6.13~14.4	NA	HtSDS, bone age, HV
Rosenfeld, R G	1998	RCT	USA	rhGH (0.375 mg/kg/week) + oxandrolone (0.0625 ~ 0.125 mg/kg/day)	rhGH	70	6.1~7.6	4.7~12.4	14	Height
Rosenfeld, R G	1986	RCT	USA	rhGH (0.375 mg/kg/week) rhGH (0.375 mg/kg/week) + oxandrolone (0.125 mg/kg/day)	No treatment oxandrolone (0.125 mg/kg/day)	70	1	4.7~12.4	NA	Bone age, HV
Gault, E J	2011	RCT	UK	rhGH (10 mg/m^2^/week) + oxandrolone (0.05 mg/kg/day)	rhGH (10 mg/m^2^/week) + placebo	106	6.2	7~13	12	Final height HtSDS
Stahnke, N	2002	RCT	German	rhGH (0.2~0.31 mg/kg/week) + oxandrolone (0.05~0.1 mg/kg/day)	rhGH (0.2~0.31 mg/kg/week)	91	4.8~5.4	10.2~10.5	14.9	HtSDS, bone age, final height
Zegar, M	2011	RCT	USA	rhGH (0.3 mg/kg/week) + oxandrolone (0.06 mg/kg/week)	rhGH + placebo (0.3 mg/kg/week)	76	4	10~14.9	NA	HtSDS

### Effects of rhGH alone on height outcome

**Figure 2:**
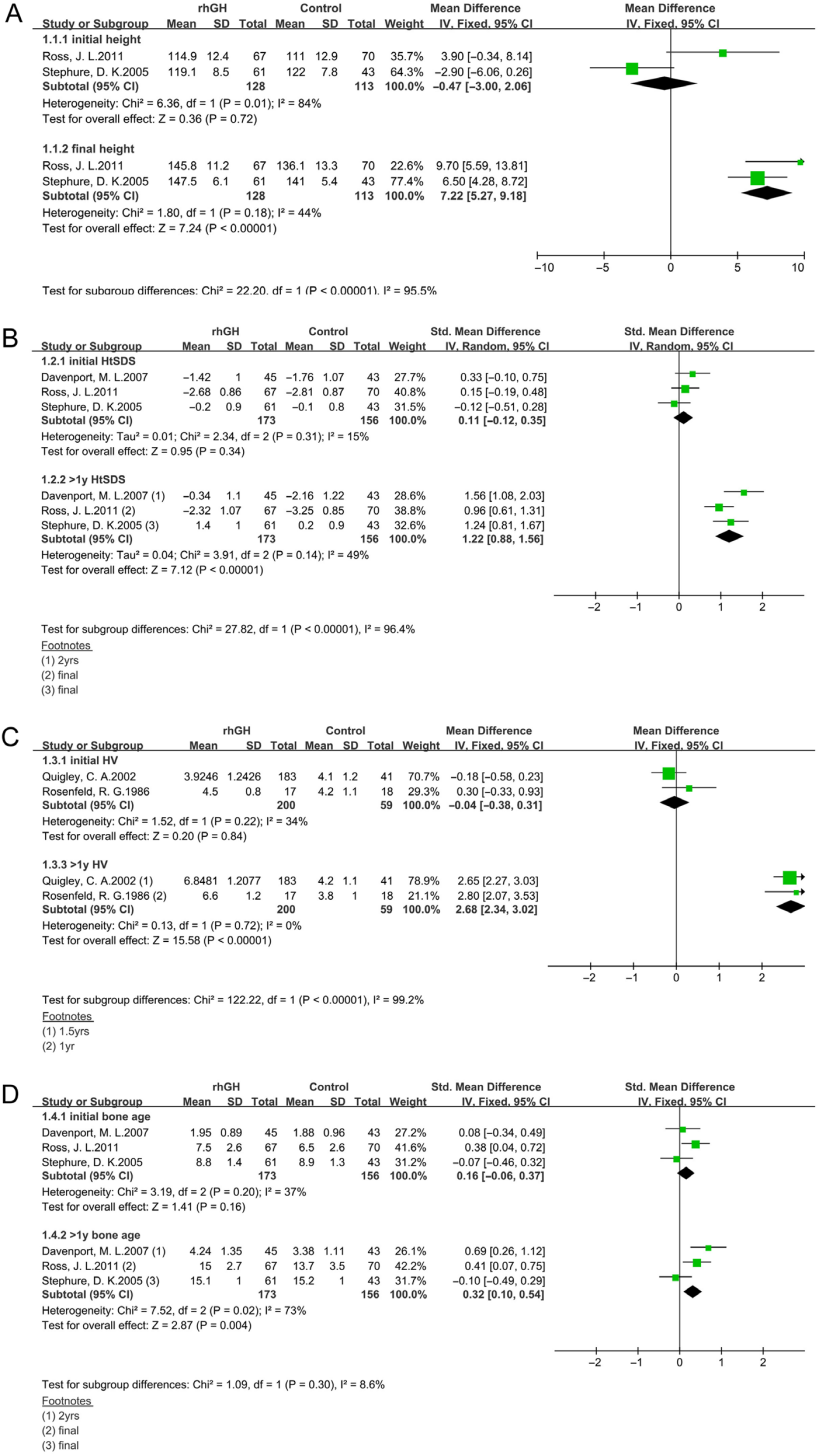
Forest plot of height outcomes in rhGH alone therapy vs control in TS girls: (A) height, (B) HtSDS, (C) HV, (D) bone age. HtSDS, height standard deviation; HV, height velocity.

We identified five studies (10, 17, 32, 33) investigating the effects of rhGH alone on height outcome (Fig. 2). Among those, two studies (10, 33) provided information on final height, HtSDS and bone age outcomes, and one study (17) did not report information on final height outcome. There were no significant differences in initial height in 2 studies (10, 33) and initial HtSDS in 3 studies (10, 17, 33), While after rhGH treatment, the overall analysis revealed significant difference in final height and HtSDS comparing the GH and control groups (mean difference(MD) = 7.22 cm, 95% CI 5.27–9.18, *P* < 0.001, *I*2 = 44%; *P* = 0.18, final height; standardized mean difference (SMD) = 1.22, 95% CI 0.88–1.56, *P* < 0.001, *I* ^2^ = 49%; *P* = 0.14, HtSDS). Similar to HV, no significant difference was seen in initial HV in two studies (32, 36); however, the result showed a higher HV in GH group than in controls (MD 2.68 cm/year; 95% CI 2.34, 3.02; *P* < 0.001, *I*2 = 0%; *P* = 0.72). Three studies (10, 17, 33) showed no significant difference in initial bone age between the two groups. After the 1-year GH treatment, there was a small advance for rhGH-treated subjects (SMD 0.32 year; 95% CI 0.1, 0.54; *P* = 0.004, *I*2 = 73%; *P* = 0.02). There was significant heterogeneity, therefore, a random-effects model of analysis was used.

### Effects of rhGH plus OX combined therapy on height outcome

**Figure 3:**
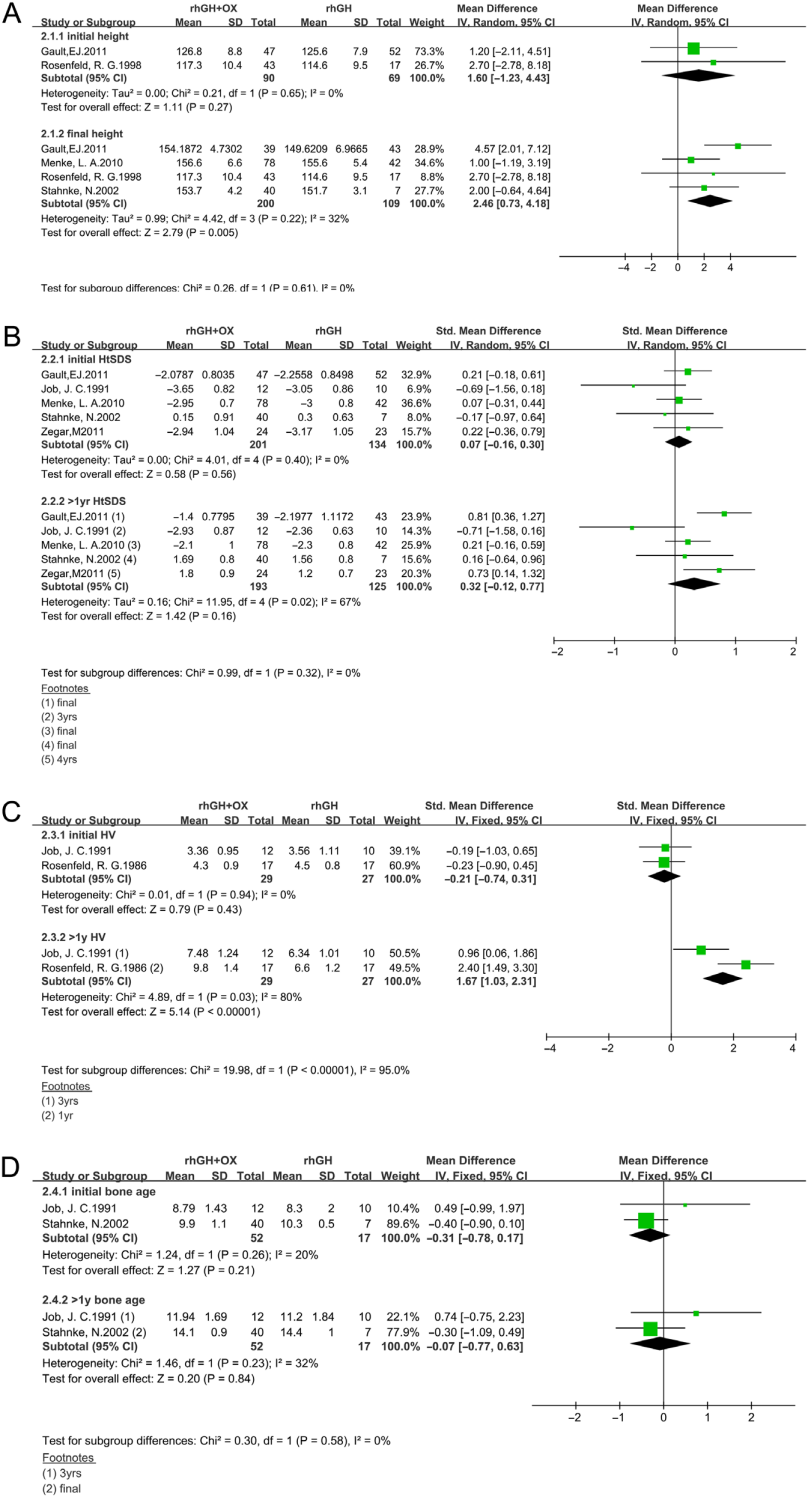
Forest plot of height outcomes in rhGH/OX combination therapy vs rhGH alone therapy in TS girls. (A) Height, (B) HtSDS, (C) HV, (D) bone age. HtSDS, height standard deviation; HV, height velocity.

As shown in Fig. 3, seven studies were included in the analysis (13, 14, 16, 31, 34, 35, 36). No significant difference was found in initial height, HtSDS, HV and bone age between the rhGH/OX combined and rhGH alone groups (*P* > 0.05). Four studies (14, 16, 34, 35) showed that girls in the combination therapy group ended up taller than girls in the rhGH alone group. Girls in the combination therapy group grew 2.46 cm taller in final height than those in the rhGH alone group (MD 2.46 cm; 95% CI 0.73, 4.18; *P* = 0.005, *I*2 = 32%; *P* = 0.22), although baseline height was similar (MD 1.6 cm; 95% CI −1.23, 4.43; *P* = 0.27). In parallel with the increases in final height, rhGH with OX-treated subjects had significantly greater increases in HV than did the rhGH alone group (SMD 1.67 cm/year; 95% CI 1.03, 2.31; *P* < 0.03, *I*2 = 80%; *P* < 0.001). There was significant heterogeneity; therefore, a random-effects model of analysis was used. Meanwhile, we also observed the effects of rhGH in combination with OX on bone age and HtSDS; however, no significant difference was seen between the two groups after treatment (*P* > 0.05).

### Risk of bias and quality of evidence assessment

**Figure 4:**
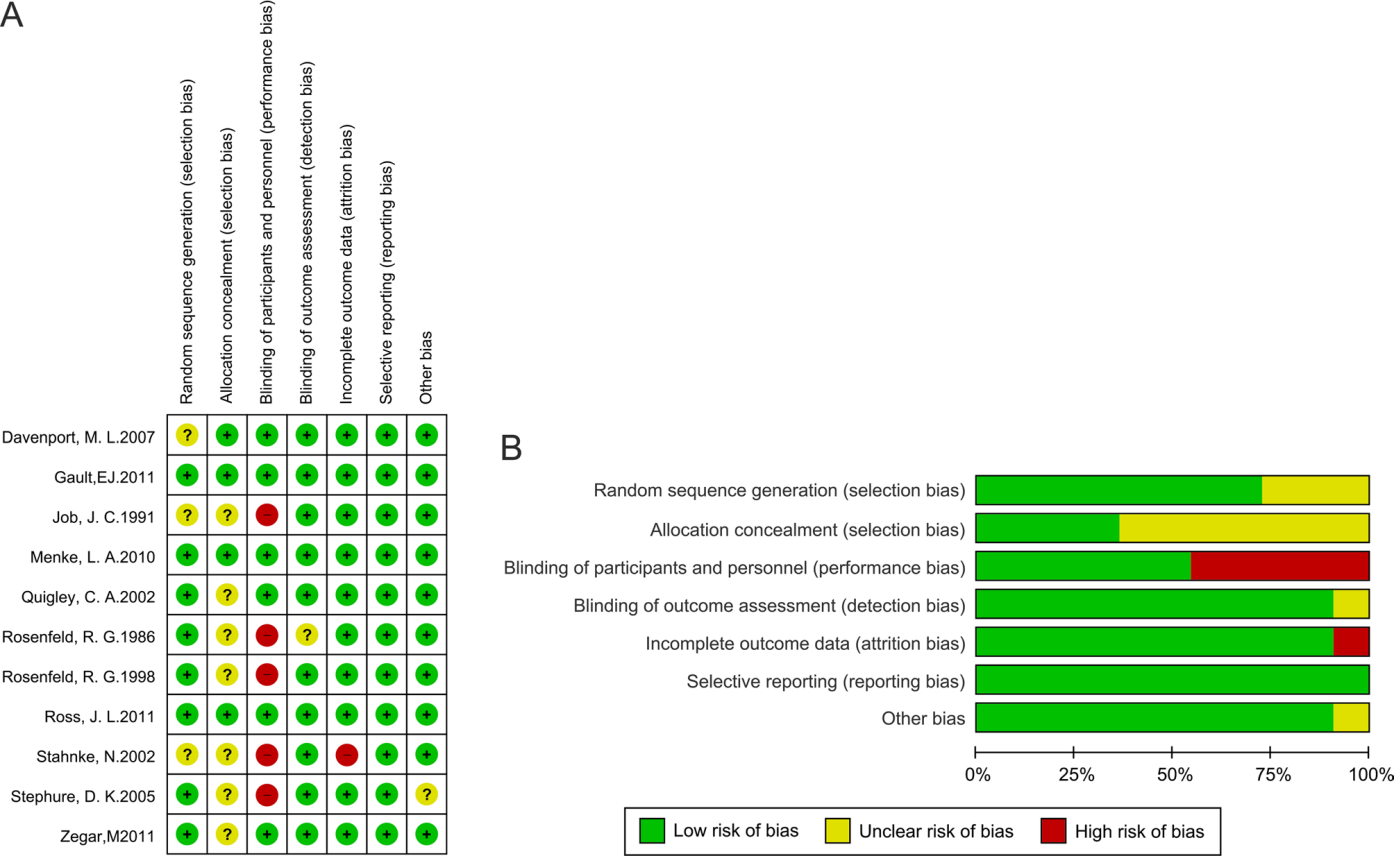
Risk of bias assessment. (A) The quality assessment for each included study as ‘risk of bias summary.’ (B) Outcomes presented as percentages across all meta-analyzed studies, each depicted as a ‘risk of bias graph’. (A) Randomized? (B) Allocation concealment? (C) Blinding? (D) Incomplete outcome data addressed? (E): Free of selective reporting? (F) Free of other bias?

As shown in Fig. 4, all studies mentioned randomization, but eight studies described the method used for sequence generation (10, 13, 14, 32, 33, 34, 35, 36). Five studies reported allocation concealment (17, 18, 33, 34, 35). Six studies described blinding of participants (13, 17, 32, 33, 34, 35), and ten studies had blinding of outcome assessors (10, 13, 14, 16, 17, 31, 32, 33, 34, 35). Ten studies showed incomplete outcome data (10, 13, 14, 17, 31, 32, 33, 34, 35, 36). Quality of evidence was assessed per outcome (Table 2) using the GRADE guidelines. Imprecision was therefore assessed by comparing the findings of the trials.

**Table 2:** GRADE evidence profile.

Outcome (number of RCTs)	Quality assessment					Quality of evidence
	Limitations	Inconsistency	Indirectness	Imprecision	Publication bias	
rhGH vs control						
Height						
Initial height	No serious limitation	serious (heterogeneity)	No serious indirectness	No serious imprecision	Undetected	⊕⊕⊕ Moderate
Final height	No serious limitations	No serious inconsistency	No serious indirectness	No serious imprecision	Undetected	⊕⊕⊕⊕ High
HtSDS						
Baseline	No serious limitations	No serious inconsistency	No serious indirectness	No serious imprecision	Undetected	⊕⊕⊕⊕High
After 1 year	No serious limitations	No serious inconsistency	No serious indirectness	No serious imprecision	Undetected	⊕⊕⊕⊕ High
Bone age						
Baseline	No serious limitations	No serious inconsistency	No serious indirectness	No serious imprecision	Undetected	⊕⊕⊕⊕ High
After 1 year	No serious limitations	serious (heterogeneity)	No serious indirectness	No serious imprecision	Undetected	⊕⊕⊕ Moderate
HV						
Baseline	No serious limitations	No serious inconsistency	No serious indirectness	No serious imprecision	Undetected	⊕⊕⊕⊕ High
After 1 year	No serious limitations	No serious inconsistency	No serious indirectness	No serious imprecision	Undetected	⊕⊕⊕⊕ High
rhGH + OX vs rhGH						
Height						
Initial height	No serious limitations	No serious inconsistency	No serious indirectness	No serious imprecision	Undetected	⊕⊕⊕⊕ High
Final height	No serious limitations	No serious inconsistency	No serious indirectness	No serious imprecision	Undetected	⊕⊕⊕⊕ High
HtSDS						
Baseline	No serious limitations	No serious inconsistency	No serious indirectness	No serious imprecision	Undetected	⊕⊕⊕⊕ High
After 1 year	No serious limitations	serious (heterogeneity)	No serious indirectness	No serious imprecision	Undetected	⊕⊕⊕ Moderate
Bone age						
Baseline	No serious limitations	No serious inconsistency	No serious indirectness	No serious imprecision	Undetected	⊕⊕⊕⊕ High
After 1 year	No serious limitations	No serious inconsistency	No serious indirectness	No serious imprecision	Undetected	⊕⊕⊕⊕ High
HV						
Baseline	No serious limitations	No serious inconsistency	No serious indirectness	No serious imprecision	Undetected	⊕⊕⊕⊕ High
After 1 year	No serious limitations	serious (heterogeneity)	No serious indirectness	No serious imprecision	Undetected	⊕⊕⊕ Moderate

## Discussion

We have performed a meta-analysis of clinical studies investigating the effect of rhGH therapy in TS patients. The current study was focused on a stringent evaluation of the efficacy of rhGH alone or with concomitant use of oxandrolone in TS. For this reason was limited to randomized controlled trials.

Our present findings suggest that rhGH alone or with concomitant use of oxandrolone therapy has beneficial effects on final height of TS patients. In girls receiving rhGH alone, there was an increase in final height, HtSDS, HV and bone age after treatment. rhGH therapy alone increases final height by 7.22 cm. The present findings regarding height outcome in rhGH-treated populations are consistent with the meta-analyses of Baxter *et al*. (37). They suggested rhGH therapy was effective in improving growth. However, the study of Baxter *et al*. included four RCTs. Among these, only one trial reported final height and two trials reported growth outcomes. Moreover, they did not explore the effect of rhGH/OX combination therapy. The evidence from previous studies has been updated by including new trials, and for the first time, the impact of rhGH/OX combination therapy on height outcome was observed, and baseline height, HtSDS, HV and bone age status have also been taken into account.

Furthermore, we found rhGH/OX-treated girls appeared more elevated final height and fasted HV than rhGH-treated girls. rhGH/OX combination therapy increases final height by 2.46 cm. High heterogeneity was found in HV performed in our study. Since only two studies (31, 36) were included, subgroup analyses or meta-regression, could not be performed. This heterogeneity may be due to the inherent differences in duration and dose of treatment. Moreover, no significant changes in HtSDS and bone age were seen between two groups. HtSDS gives an indication of height relative to other children of the same age or relative to other adults or relative to a population with TS. Since the studies used different assays for HtSDS, this might influence any potential trend. Moreover, two trials (13, 31) provided information of HtSDS after 3 years rhGH treatment; however, three trials (16, 34, 35) provided final HtSDS.

Another concern may come from the age and dose for initiation of therapy and the duration of therapy. To date, the optimal age for initiation of rhGH therapy for young children has not been established. Age for initiation of therapy of this meta-analysis has had wide variations (from 9 months to 10.2 years). With respect to rhGH dose, treatment was generally administered at fixed doses, ranging from physiological (0.2 mg/kg/week) to supraphysiological (0.43 mg/kg/week) doses. Similar to rhGH, OX dose ranged from 0.03 to 0.125 mg/kg/day doses. Only small studies compared various rhGH dosages (9, 38, 39); therefore, we were not able to meta-analyze dose–response in TS girls.

A limitation of the present meta-analysis is the relatively small number of available studies and the lack of sufficient data on final height. Subgroup analyses or meta-regression, using study level covariates, could not be performed because of the relatively small number of available investigations. Although a blinded study is less likely to be done for rhGH is approved and standard practice for girls with TS, larger studies of longer duration using a blinded, placebo-controlled design are needed to clarify these issues. Second, the trial reporting final height had lost some participants at the time of reporting. It is possible that treated girls who have not achieved final height would be more likely to be excluded. Similarly, girls in the control group who were growing more slowly might be more likely to leave the trial.

In conclusion, the findings of the present meta-analysis suggest that rhGH alone or with the concomitant use of oxandrolone therapy in TS patients may improve final height.

## Declaration of interest

The authors declare that there is no conflict of interest that could be perceived as prejudicing the impartiality of the research reported.

## Funding

This work did not receive any specific grant from any funding agency in the public, commercial or not-for-profit sector.

## Author contribution statement

P L and F C conceived and designed the experiments. P L, L X and H L carried out the experiments and calculations. P L and F C wrote and edited the paper. All authors reviewed the manuscript.
